# Supplementary material for: Molecular Dynamics Simulations of γ‐Belite(010)–Water Interfaces With High‐Dimensional Neural Network Potentials
Source: Chemistry. 2026 Apr 9;32(24):e70956. doi: 10.1002/chem.70956 (PMC13290424; doi:10.1002/chem.70956)
Supplement: Supplementary file 1 — Supporting File: chem70956‐sup‐0001‐SuppMat.pdf. [file CHEM-32-e70956-s001.pdf]

Supporting Information: Molecular Dynamics Simulations of γ-Belite(010)-Water Interfaces with High-Dimensional Neural Network Potentials

Bernadeta Prus and Jörg Behler*
Lehrstuhl für Theoretische Chemie II, Ruhr-Universität Bochum, 44780 Bochum, Germany, and Atomistic Simulations, Research Center Chemical Sciences and Sustainability, Research Alliance Ruhr, 44780 Bochum, Germany
(Dated: December 8, 2025)

A. DENSITY FUNCTIONAL THEORY CALCULATIONS

The DFT reference calculations for training the high-dimensional neural network potentials (HDNNP) have been performed using the all-electron FHI-aims code (version 221103)[1]. The RPBE functional[2] has been chosen to describe electronic exchange and correlation in combination with light settings for the numerical atomic orbital basis set and integration grids. For the periodic systems, a **k**-point density of 4/Å^{-1} was chosen resulting in a **k**-point grid of 5×3×4 for the 28 atom bulk unit cell of γ-belite with dimensions of 5.134×11.211×6.753Å^3[3]. For the slab calculations, the **k**-point grids have been adjusted to maintain the same density.

Before training the HDNNP, dispersion corrections have been added to the energies and forces employing the D3 method of Grimme using the DFT–D3 v.3.1 code[4] with zero damping, since RPBE-D3 has been demonstrated to provide a good description of liquid water[5–8] and also of bulk cementitious minerals[9]. The DFT-based structural optimizations were carried out through the Atomic Simulation Environment (ASE)[10] with a convergence criterion of 0.05 eV/Å, no symmetry constraints were applied.

B. MOLECULAR DYNAMICS SIMULATIONS

Molecular Dynamics (MD) simulations were carried out with the Large-scale Atomic/Molecular Massively Parallel Simulator (LAMMPS - version from 15 Jun 2023)[11] in combination with the Neural Network Potential Package (n2p2 v2.2.0)[12], which allows to use energies, forces and the stress tensor provided by HDNNPs trained with *RuNNer*[13,14]. Depending on the property of interest, the MD simulations were run in the NPT or NVT ensemble. A Nosé–Hoover thermostat (300 K) and/or barostat (1 bar) was applied, with the barostat coupled only to the surface normal direction for simulations of solid-liquid interfaces to maintain the bulk lattice constant in the interior of the γ-belite slabs. The MD time step was set to 1 fs for bulk γ-belite and to 0.5 fs for bulk water as well as the solid-liquid interface structures. In the case of solid-liquid interfaces, to prevent movement of the slab in the x and y directions, the total momentum of the central part of the belite slab with a diameter of 9 Å was fixed in those directions.

MD simulations of bulk γ-belite were performed for (3×2×3) supercells containing 756 atoms in the NPT ensemble for 1 ns. For MD simulations of the water/belite interfaces initial slabs were prepared as (4×4) supercells of the surface unit cells in vacuum described above. They were separated by a water film with a diameter of about 60 Å containing 576 molecules. In total, each studied interface system contains 4416 atoms. Before being placed in the vacuum between the slab surfaces, the aqueous regions were equilibrated in the NVT ensemble for 25 ps by HDNNP-driven MD simulations at 300 K employing a density of 0.998 g/cm^3. The water films were then inserted in the vacuum regions of the slabs with an initial vertical separation of about 2.5 Å between the water region and both surfaces. To close this gap, the water-belite interfaces were then equilibrated for 1 ns in the NPT ensemble. After this NPT simulation, for the central part of the water region with 25 Å diameter, which is about 20 Å away from both surfaces, a density of about 0.92 g/cm^3 was found, which is very close to the RPBE+D3 equilibrium density of bulk water[7]. This is in agreement with previous studies on other solid-liquid interfaces showing that water films of this thickness adopt a bulk-like water structure in the center[15]. The properties of the interfaces were then determined in 1 ns MD simulations in the NVT ensemble.

C. RADIAL DISTRIBUTION FUNCTIONS

Based on the HDNNP-driven NPT MD simulation of the (3 × 2 × 3) supercell, the bulk γ-belite radial distribution function was calculated at 300 K (Fig S1). The strong first Si-O peak is related to the four oxygen atoms coordinating Si atoms in a tetrahedral arrangement. The first coordination shell of the calcium atoms can be found in a range from 2.1 to 2.9 Å with a maximum at 2.4 Å.

* joerg.behler@rub.de

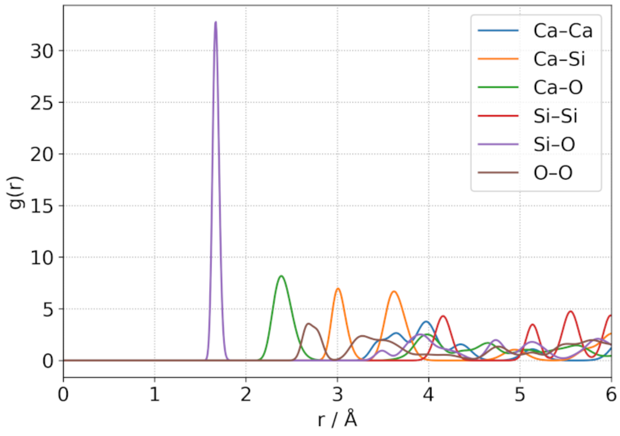

FIG. S1. Radial distribution functions in bulk γ-belite obtained by MD simulations of a $(3 \times 2 \times 3)$ supercell in the NPT ensemble at 300 K.

D. REFERENCE DATA SET CONSTRUCTION

The reference data set consists of three parts: bulk γ-belite, bulk liquid water, and solid-liquid interface structures. The reference data set composition is compiled in Table SI.

TABLE SI. Composition of the reference data set. Given is the number of structures together with the respective number of atoms per structure for the three types of systems.

Type	Atoms	Structures
Bulk γ-belite		
	28	2522
	224	839
Total		3361
Water		
	24	932
	30	222
	36	556
	48	3106
	96	900
	192	920
	384	120
Total		6756
Water-belite interfaces		
	208	9013
	304	4313
	512	594
	1620	347
Total		14267
Sum over all types		24384

For bulk liquid water, geometries were taken from previous work[7], recalculated with the DFT settings described above. The high-pressure ice structures in the dataset were not included in the present work. The initial bulk γ-belite structures were prepared based on random atomic displacements around the experimental and DFT-optimized geometries. In addition, the system was expanded and compressed by changing the unit cell parameters while keeping fractional coordinates of the atoms fixed. The initial reference data set of 971 geometries was used as a starting point for the iterative extension of the data set by an active learning approach - RuNNerActiveLearn[16,17] - using MD simulations in the NPT ensemble at a pressure of 1 bar and a temperature range of 100-500 K. The final reference data set contains 3361 bulk γ-Belite structures (2522 $1\times1\times1$ and 839 $2\times2\times2$ supercells).

The third part of the reference data set contains interface structures of both investigated terminations as well as 32, 64, and 96 water molecules. Additionally, for T2 largers systems with 288 water molecules were prepared. Interfaces were constructed based on γ-belite slabs of different thicknesses. The initial interfacial structures were prepared with the following method: first, periodic cells laterally matching the dimensions of the surface slabs containing 32 water molecules of density equal to 0.998 g/cm^3 were equilibrated through NVT MD simulations at 300 K using a preliminary HDNNP for water trained to the bulk liquid water data only. Ten uncorrelated water structures were then inserted in the vacuum regions of optimized surfaces at a distance between 2.3-2.7 Å. Prepared in this way, 50 interfaces were subsequently optimized using DFT with a force convergence criterion of 0.05 eV/Å. From the obtained optimization pathways every 25th structure was included in the initial interface reference data set containing 522 structures for T3 and 513 for T2. The active learning procedure for the interfaces has been started for the most stable T3 termination, based on NPT simulations at a pressure of 1 bar along the z-direction perpendicular to the surface and a temperature range of 275-375 K. From this point, the HDNNP was trained based on a combined reference data set, containing bulk water, bulk γ-belite, and the interface structures. As soon as the MD simulations became stable for about 5-10 ps, the T2 termination of the (010) surface was also included in the active learning, which was then continued for both surfaces.

For all types of geometries, only structures with absoluted forces smaller than 10 eV/Å were included in the reference data set.

E. ATOM-CENTERED SYMMETRY FUNCTIONS

The local atomic environments with a cutoff radius of 6 Å are described by 188, 191, 167, and 165 atom-centered symmetry functions (ACSFs)[18] for hydrogen, oxygen, calcium, and silicon atoms, respectively. Ra-

dial ACSF have been contructed for all atom pairs using the set of η values 0.00000000, 0.01396710, 0.06161784, and 0.48552766 bohr^{-2}. For Ca-Si, Ca-Ca, Si-Si, and Si-Ca, the symmetry function for the highest η value has been omitted. Moreover, a second set of shifted radial symmetry functions with a maximum at 0.9 bohr and the η values 1.0000, 3.5845, 6.1690, and 8.7535 bohr^{-2} has been included. Here, the ACSF with η equal to 1.0 bohr^{-2} has been omitted for Ca-Si, Ca-Ca, Si-Si, and Si-Ca atom pairs. For the generation of an angular symmetry function, the starting point is a pool of combinations of λ={-1,1}, ζ={1,2,4,8}, and η={0,0.06161784}. From this pool the following ACSFs were removed for specific element triples, as the range of values has been small:

Element triple	η	λ	ζ
H-Si-Si	0.06161784	-1.0	8.0
H-Si-Si	0.06161784	1.0	2.0, 4,0 , 8.0
O-Si-Si	0.06161784	1.0	8.0
Si-H-Si	0.06161784	-1.0	1.0, 2.0, 4.0, 8.0
Si-O-Si	0.06161784	-1.0	4.0, 8.0
Si-O-Si	0.00000000	-1.0	4.0, 8.0
Si-Si-Si	0.00000000	-1.0	4.0, 8.0
Si-Si-Si	0.06161784	-1.0	1.0, 2.0, 4.0, 8.0
Si-Si-Si	0.06161784	1.0	1.0, 2.0, 4.0, 8.0
Si-Si-Ca	0.06161784	-1.0	1.0, 2.0, 4.0, 8.0
Si-Si-Ca	0.06161784	1.0	8.0
Si-Ca-Ca	0.06161784	-1.0	4.0, 8.0
Ca-H-Si	0.06161784	-1.0	8.0
Ca-H-Ca	0.06161784	-1.0	4.0, 8.0
Ca-O-Si	0.06161784	-1.0	8.0
Ca-O-Ca	0.06161784	-1.0	4.0, 8.0
Ca-Si-Si	0.06161784	1.0	2.0, 4.0, 8.0
Ca-Si-Ca	0.06161784	-1.0	4.0, 8.0
Ca-Ca-Ca	0.06161784	-1.0	1.0, 2.0, 4.0, 8.0
Ca-Ca-Ca	0.06161784	1.0	4.0, 8.0

F. *RuNNer* SETTINGS

The input settings used for training HDNNPs with *RuNNer* are listed in Table SII. The different HDNNPs used for active learning have been obtained by using different random numbers for the initial neural network weights as well as the splitting into a training and test set.

G. ENERGY AND FORCE ERROR PLOTS

TABLE SII. Settings of the *RuNNer* input file used for training the HDNNPs.

**Keyword**	**Value**
nn_type_short	1
random_number_type	5
random_seed	316
number_of_elements	4
elements	H O Si Ca
cutoff_type	1
use_short_nn	
global_hidden_layers_short	2
global_nodes_short	15 15
global_activation_short	ttl
test_fraction	0.1
epochs	30
repeated_energy_update	
mix_all_points	
scale_symmetry_functions	
center_symmetry_functions	
fitting_unit	eV
precondition_weights	
use_short_forces	
kalman_lamda_short	0.98
kalman_nue_short	0.9987
short_energy_fraction	0.01
short_force_fraction	1.0
force_scale_update	4.0
nguyen_widrow_weights_short	
short_force_error_threshold	1.0
short_energy_error_threshold	0.1
remove_atom_energies	
atom_energy	Ca -682.06533
atom_energy	Si -290.32622
atom_energy	O -75.18080
atom_energy	H -0.45891

Figures S2 and S3 present the deviations between the HDNNP and the DFT-D3 reference energies as a function of the reference energy for the training and test data sets, respectively. Plots are split in multiple segments due to the large offsets in total energies for different parts of the reference data set due to different stoichiometries. For both data sets the difference between the predicted and reference energies for the majority of structures is smaller than 1 meV/atom.

Figures S4 and S4 present the force component deviations for the training and test data sets. For forces in the range from -2.5 to 2.5 eV/ Å very rarely differences between the HDNNP and reference forces of up to 1 eV/ Å are observed. However, in the full reference data

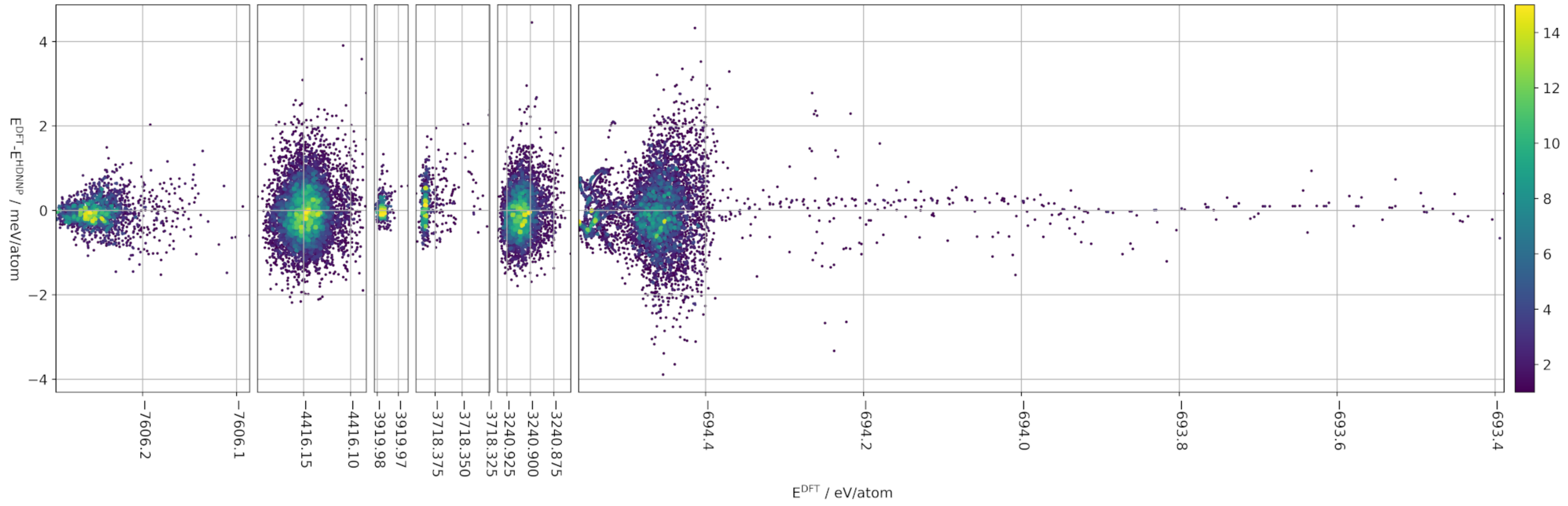

FIG. S2. Energy error plots showing the deviations between the HDNNP predictions and the DFT reference values for the training data set. The data points are colored based on their relative density, highlighting regions of higher data population.

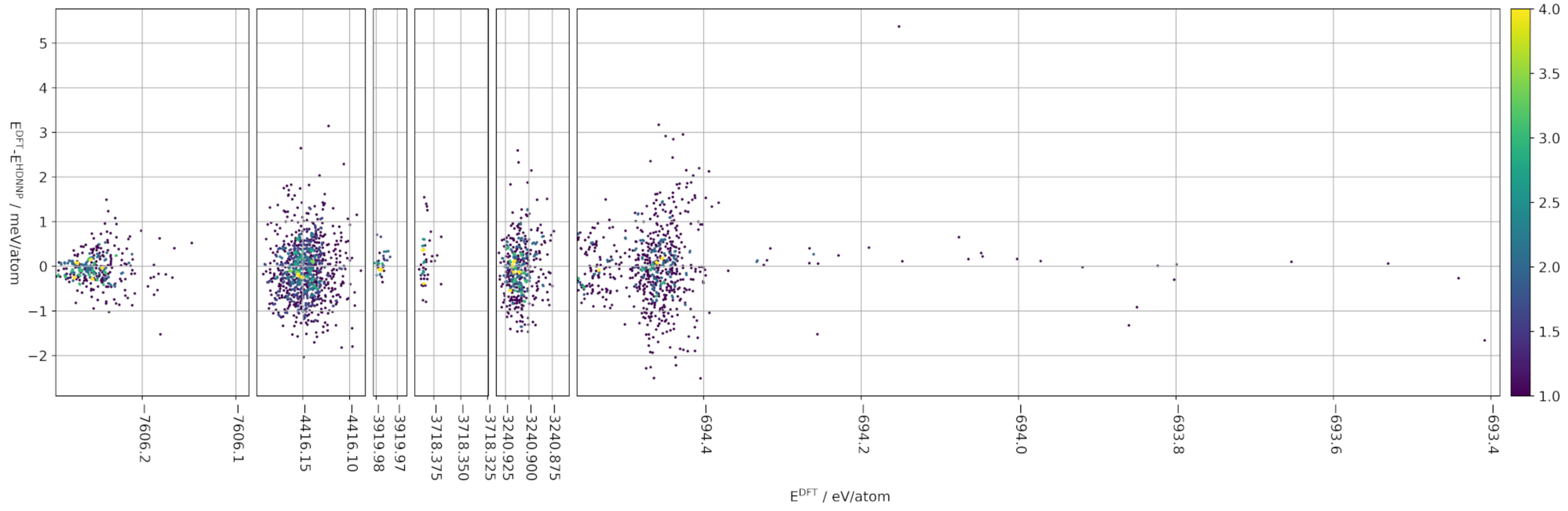

FIG. S3. Energy error plots showing the deviations between the HDNNP predictions and the DFT reference values for the test data set. The data points are colored based on their relative density, highlighting regions of higher data population.

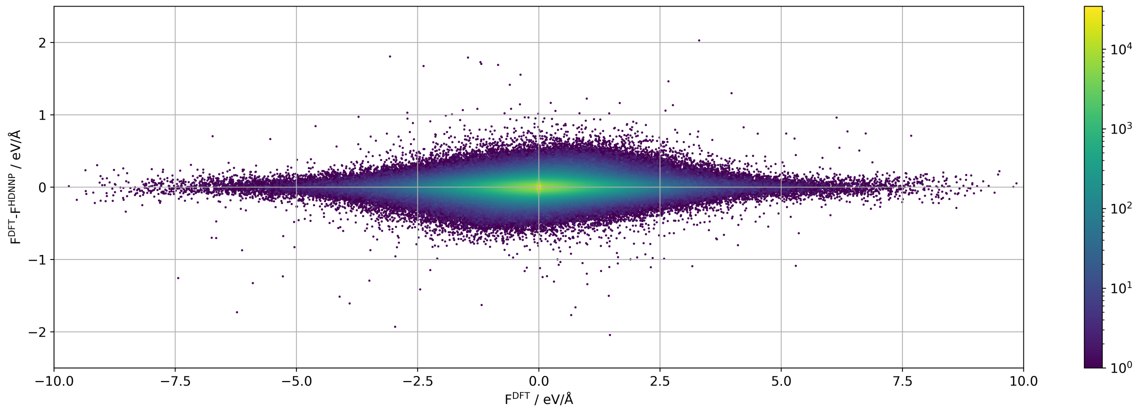

FIG. S4. Force error plots showing the deviations between the HDNNP predictions and the DFT reference values for the training data set. The data points are colored based on their relative density, highlighting regions of higher data population.

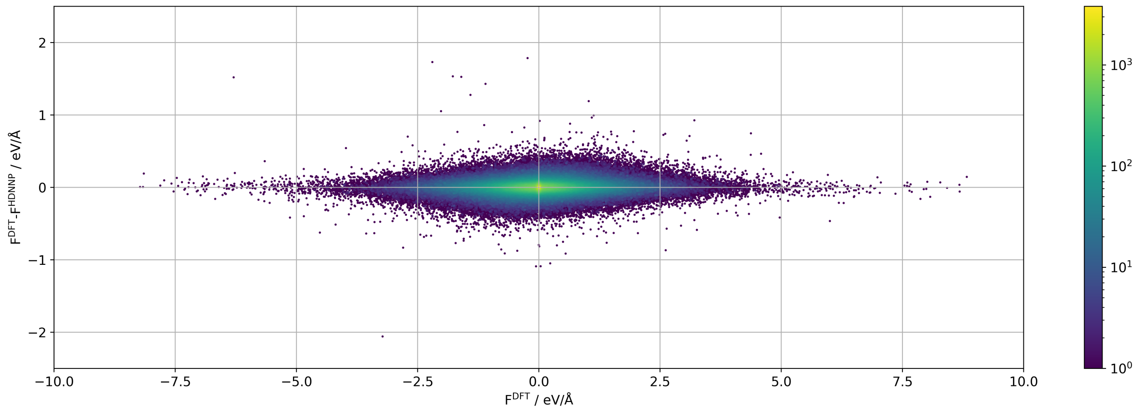

FIG. S5. Force error plots showing the deviations between the HDNNP predictions and the DFT reference values for the test data set. The data points are colored based on their relative density, highlighting regions of higher data population.

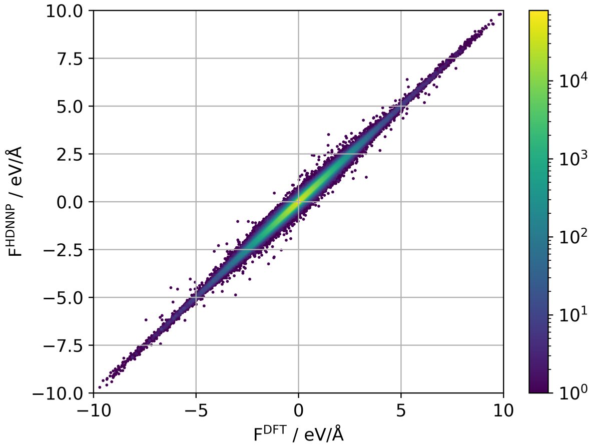

FIG. S6. Force error plots showing the correlation between the HDNNP predictions and the DFT reference values for the training data set. The data points are colored based on their relative density, highlighting regions of higher data population.

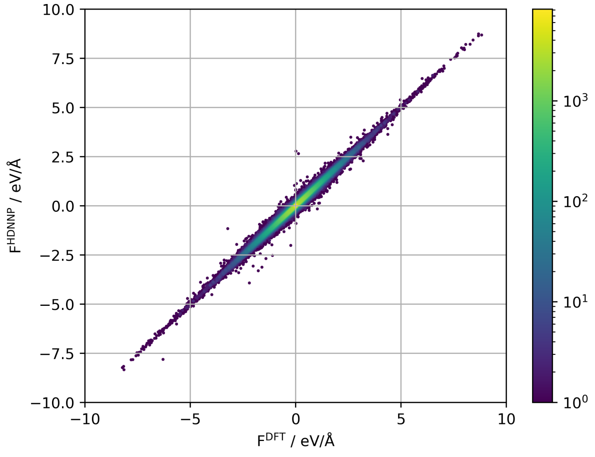

FIG. S7. Force error plots showing the correlation between the HDNNP predictions and the DFT reference values for the test data set. The data points are colored based on their relative density, highlighting regions of higher data population.

set containing 14,453,784 force components, only 1.5% of the force components have an absolute value of force deviations larger than the force 3*RMSE of the test set (3*RMSE(F) = 0.2052 eV/Å). Additionally, Figures S6 and S7 present the correlation between the HDNNP-predicted force components and their reference values.

H. EQUILIBRATION OF THE MD SIMULATIONS

Figures S8 and S9 show the time evolution of different oxygen species at T2 and T3 during the initial equilibration of the system by MD in the NPT ensemble at 300 K. After about 1 ns simulation time equilibrium has been reached.

[1] V. Blum, R. Gehrke, and F. e. a. Hanke, Computer Physics Communications **180**, 2175 (2009).
[2] B. Hammer, L. B. Hansen, and J. K. Nørskov, Physical Review B **59**, 7413 (1999).
[3] E. R. Gobechiya, N. A. Yamnova, A. E. Zadov, and V. M. Gazeev, Crystallography Reports **53**, 404 (2008).
[4] S. Grimme, J. Antony, S. Ehrlich, and H. Krieg, The Journal of Chemical Physics **132**, 154104 (2010).
[5] T. Morawietz and J. Behler, The Journal of Physical Chemistry A **117**, 7356 (2013).
[6] K. Forster-Tonigold and A. Groß, The Journal of Chemical Physics **141**, 064501 (2014).
[7] T. Morawietz, A. Singraber, C. Dellago, and J. Behler, Proceedings of the National Academy of Sciences **113**, 8368 (2016).
[8] K. N. Lausch, R. El Haouari, D. Trzewik, and J. Behler, The Journal of Chemical Physics **163**, 034101 (2025).
[9] D. Prasad and N. Mitra, The Journal of Physical Chemistry C **126**, 11265 (2022).
[10] A. Hjorth Larsen, J. Jørgen Mortensen, and J. e. a. Blomqvist, Journal of Physics: Condensed Matter **29**, 273002 (2017).
[11] A. P. Thompson, H. M. Aktulga, and R. e. a. Berger, Computer Physics Communications **271**, 108171 (2022).
[12] A. Singraber, J. Behler, and C. Dellago, Journal of Chemical Theory and Computation **15**, 1827 (2019).
[13] J. Behler, International Journal of Quantum Chemistry **115**, 1032 (2015).
[14] J. Behler, Angewandte Chemie International Edition **56**, 12828 (2017).
[15] S. Kondati Natarajan and J. Behler, Physical Chemistry Chemical Physics **18**, 28704 (2016).
[16] M. Eckhoff and J. Behler, Journal of Chemical Theory and Computation **15**, 3793 (2019).
[17] M. Eckhoff and J. Behler, npj Computational Materials **7**, 170 (2021).
[18] J. Behler, The Journal of Chemical Physics **134**, 074106 (2011).

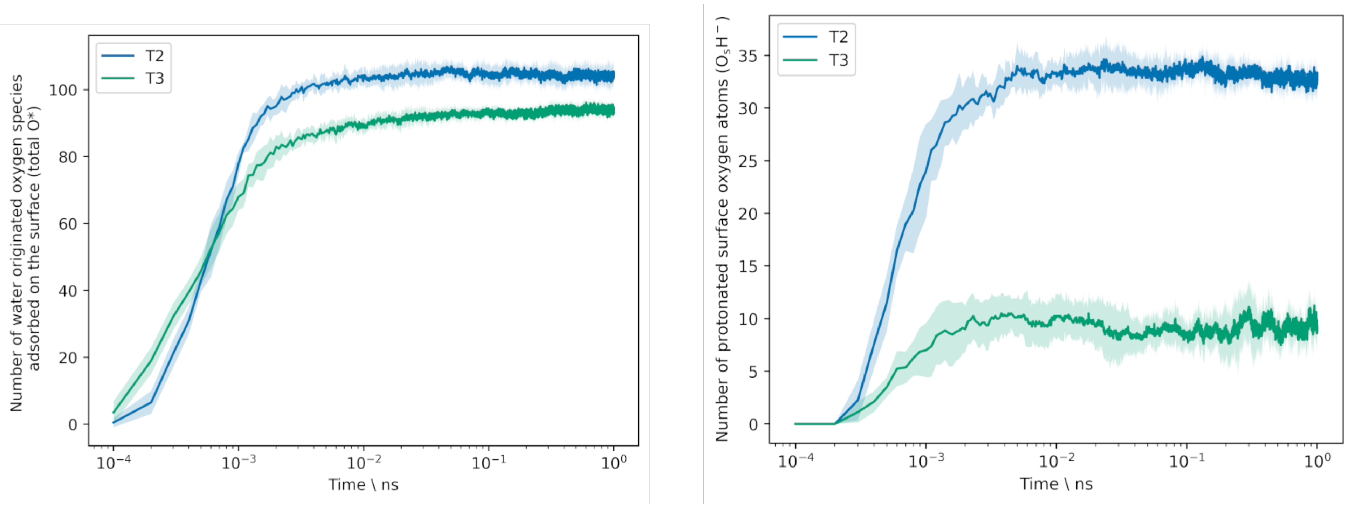

FIG. S8. Number of average adsorbed oxygen atoms (a) and protonated surface oxygen atoms (b) during *NPT* MD simulations at 300 K of the T2 and T3 terminations, plotted with standard deviation over 8 trajectories.

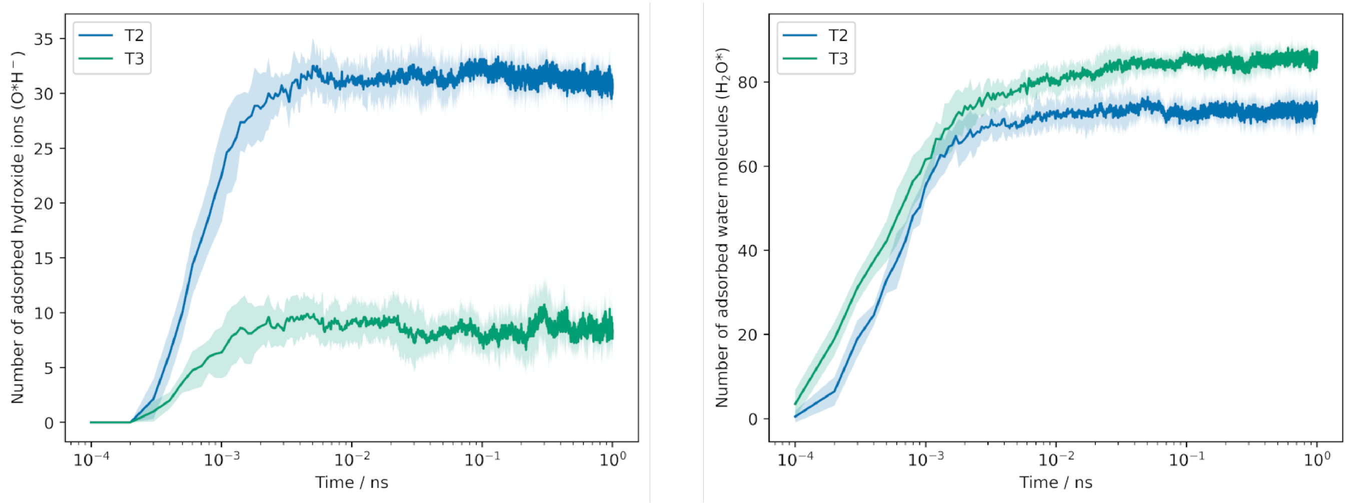

FIG. S9. Number of average adsorbed oxygen atoms with distinction between hydroxide ions (a) and water molecules (b) during *NPT* MD simulations at 300 K of the T2 and T3 terminations, plotted with standard deviation over 8 trajectories.
